# Retraction: Enterococcal Infective Endocarditis following Periodontal Disease in Dogs

**DOI:** 10.1371/journal.pone.0259200

**Published:** 2021-10-22

**Authors:** The PLOS ONE Editors

After this article [1] was published, concerns were raised about aspects of the methods, results, and conclusions. In light of the issues raised, *PLOS ONE* reassessed the article with input from two Academic Editors. The following issues were identified and/or confirmed in the post-publication assessment:

1. Questions were raised as to the validity of methods used to diagnose infective endocarditis (IE) and periodontal disease (PD), and it was raised that visual diagnosis may result in over-diagnosis of endocarditis since histological examination is needed to distinguish it from degenerative valvular disease [2, 3].
  The authors confirmed that diagnoses were based on visual observation by experienced veterinarians, as reported in [1]. No additional tests were performed to diagnose PD or IE.
  An Academic Editor noted that visual observation is not sufficient to diagnose IE, and that the reported enterococcal IE prevalence was higher than would be expected.
  The consulted Academic Editors confirmed that PD diagnosis is often done visually, but they advised that further diagnosis beyond visual assessment would be beneficial to confirm the level of PD. They also noted that additional details about the presentation and/or severity of specific PD symptoms should have been reported.
2. The consulted Academic Editors advised that the study design was not sufficient to identify which bacterial species were present in the samples, demonstrate whether the same species was identified in different samples, and clarify whether results were indicative of sample contamination or in vivo infection.
3. Per the editorial assessment, the article’s title and conclusions overstated the results.

The above issues call into question the validity of the article’s overall results and conclusions. Therefore, the *PLOS ONE* Editors retract this article.

All authors disagreed with retraction and stand by the article’s findings. In response to point 2, above, the authors noted that necropsies were performed by a trained DVM, within a maximum of 15 minutes after death to avoid autolysis and tissue contamination, using proper necropsy techniques to avoid cross-contamination. The authors also stated that at the time of the study, macrorestriction analysis using pulsed-field gel electrophoresis (PFGE) was considered an accepted technique for evaluating genomic relatedness between isolates.

The authors clarified the following in post-publication discussions:

- The related studies reported in [1, 4, 5] used overlapping samples but were based on analyses performed during different time periods, with different objectives and methods.
- There were reporting errors in [1], in the sentence, “…a total of 35 were positive for the presence of *Enterococcus* spp., 21 mouth swabs and 13 from the heart”. The study used 18 males and 14 females (as is also reported in [4]), and 34 samples tested positive for *Enterococcus* spp.
- There was a citation error in the sentence, “Considering that the same isolate was present in both the oral cavity and heart valves of dogs with PD and IE, this suggests the occurrence of enterococci dissemination between the animals’ mouth and heart, as already described in human IE cases [6].” This should instead have referenced the Okui et al. (2015) article listed as reference 7 in [1].
